# Surface Engineering on Transition Metal Dichalcogenides: In Situ Encapsulation of Metal–Organic Frameworks for Highly Humidity‐Resistant Gas Sensing at Room Temperature

**DOI:** 10.1002/advs.74614

**Published:** 2026-02-28

**Authors:** Junwei Zeng, Weicheng Jiao, You Wang, Ye Yuan, Xiaodong He, Yue Niu

**Affiliations:** ^1^ National Key Laboratory of Science and Technology on Advanced Composites in Special Environments Harbin Institute of Technology Harbin P. R. China; ^2^ School of Materials Science and Engineering Harbin Institute of Technology Harbin P. R. China; ^3^ Songshan Lake Materials Laboratory Dongguan P. R. China; ^4^ Dongguan Key Laboratory of Interdisciplinary Science for Advanced Materials and Large‐Scale Scientific Facilities School of Physical Sciences Great Bay University Dongguan P. R. China

**Keywords:** 2D materials, gas sensor, humidity resistance, metal–organic frameworks

## Abstract

For their large specific surface areas and strong affinity with water molecules, two‐dimensional (2D) material‐based gas sensors are highly susceptible to the inference of ubiquitous relative humidity (RH) fluctuance, resulting in reduced accuracy and poor stability in real‐world conditions. In this study, a metal–organic framework (MOF) encapsulation layer, 2D UiO‐66‐NH_2_, was in situ constructed on the surface of molybdenum disulfide (MoS_2_) via an aqueous synthesis method. Benefiting from the unique 2D UiO‐66‐NH_2_ morphology formed on MoS_2_ surface, the humidity‐resistant nitrogen dioxide (NO_2_) sensing performance of 2D UiO‐66‐NH_2_/MoS_2_ is dramatically enhanced within a wide RH range from 35% to 75% owing to the forceful suppression of the water molecules adsorption and condensation. It is worth mentioning that the selectivity of the material also improved as it is effectively shielded from interactions with water molecules and interfering gases. Furthermore, the practical limit of detection (LOD) for NO_2_ of the sensor reaches an ultralow concentration of 20 ppb. The surface engineering strategy we demonstrated exhibits a significant advancement in the fabrication of high‐performance, especially excellent humidity‐resistant and selective 2D material‐based gas sensors at room temperature.

## Graphical abstract

2D UiO‐66‐NH_2_/MoS_2_ was fabricated via an aqueous synthesis method to in situ encapsulation of MoS_2_ with 2D UiO‐66‐NH_2_ MOF. The humidity‐resistant NO_2_ sensing of 2D UiO‐66‐NH_2_/MoS_2_ at room temperature was accordingly enhanced by MOF encapsulation layer, along with the selectivity and stability. This strategy provided a promising route for the design of humidity‐resistant gas sensors.

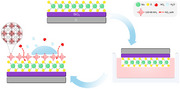

## 1 Introduction

The rapid advancement of emerging industries, particularly in artificial intelligence (AI) [1, 2], health monitoring [3, 4], and smart agriculture [5, 6], has driven significant progress in sensor technology. As a pivotal component in the Internet of Things (IoT), gas sensors, valued for their reliability and sensitivity, have been considered as an electronic nose that surpasses the limits of human olfaction. Conventional metal oxide‐based gas sensing materials must rely upon abundant active oxygen species that are commonly generated at high operating temperature (> 200 °C), probably increasing device complexity and raising safety concerns [7]. Following the discovery of graphene, other two‐dimensional (2D) materials such as transition metal dichalcogenides (TMDCs) have demonstrated noteworthy potential for stimuli‐responsive applications because of their high specific surface areas and unique electronic properties arising from their low‐dimensional structures [8, 9]. In contrast to graphene, TMDCs possess layer‐dependent bandgaps ranging from 0.2 to 3 eV [10, 11]. Besides, the widely accepted gas sensing mechanism of TMDCs depends on the charge transfer with target gases that are directly adsorbed onto the material's surface [12, 13]. These characteristics endow TMDCs with high sensitivity and room‐temperature gas sensing capabilities, making them promising alternatives to the traditional oxide metal materials in room‐temperature gas sensing applications [14].

Nonetheless, the principle enabling semiconductive gas sensors is based on the resistance alterations in different gaseous environments, which makes them vulnerable to variations in ambient conditions, such as temperature and humidity [15]. Notably, owing to their high surface‐to‐volume ratio and unique electronic transport properties, TMDCs are extremely sensitive to humidity [16]. Atmospheric water molecules exhibit lower desorption rates at room temperature, making them prone to retain on the surface of the sensing materials [17]. Moreover, the adsorbed water molecules subsequently induce proton conductivity and inhibit gas sensing interactions, both of which alter TMDCs resistance and degrade their gas sensing performance [18]. In order to minimize the effects of humidity fluctuations and achieve the desired humidity‐resistant properties, surface engineering represents a straightforward and forceful strategy, including noble metal doping [19], introduction of hydrophilic materials [20], and hydrophobic layers encapsulation [21]. However, compositions with either noble metal or hydrophilic materials still suffer from the need for elevated temperature to boost gas molecules diffusion. Despite the fact that hydrophobic layers play an intrinsic role to block water molecules from contacting the sensing materials, they typically introduce a barrier between sensing layers and target gases, which ordinarily delays the gas sensing responses at room temperature and poses challenges for further practical applications [18].

Therefore, to guarantee the superior gas sensing performance of sensing materials under fluctuating environmental relative humidity (RH), the ideal hydrophobic layer should possess a large specific surface area, well‐defined gas diffusion channels, and abundant active sites. Benefiting from their ultrahigh porosity, tailorable cavity and unique topological structure, metal–organic frameworks (MOFs) are regarded as promising materials for molecular‐sieving layers and encapsulation layers [22, 23, 24]. In the gas sensing community, several strategies have been reported. For instance, a heterostructure was fabricated by overlaying a monolayer 2D MOF, ZnTPyP, onto platinum diselenide (PtSe_2_) thus tuning the primary sensing analyte of PtSe_2_ from nitrogen dioxide (NO_2_) to hydrogen disulfide (H_2_S) [25]. Zeolitic imidazolate framework leaf (ZIF‐L) was dip‐coated on tin disulfide (SnS_2_) as a molecular‐sieving layer, realizing ultraselective H_2_S detection at 125 °C [26]. However, MOF layers integrated with 2D materials through above methods inclined to thoroughly cover the material surface, impeding the contact with the electrodes and noticeably decreasing the pristine sensing performance. Recently, ZIF‐8 and UiO‐66‐NH_2_ have been grown on graphene substrates via an innovative aqueous synthesis [27, 28]. To suppress homogeneous nucleation of MOFs in the bulk solution, it was facile to promote the in‐plane growth of 2D nuclei and slow its out‐of‐plane growth on 2D materials. Nevertheless, in situ MOFs growth on TMDCs for enhanced gas sensing, especially with highly humidity resistance, are still rarely reported.

Herein, we demonstrated for the first time an in situ encapsulation of a 2D UiO‐66‐NH_2_ MOF layer on the flat surface of mechanically exfoliated molybdenum disulfide (MoS_2_) via the aqueous synthesis method driven by van der Waals interaction. Taking advantage of the exceptional MOF morphology, the 2D UiO‐66‐NH_2_/MoS_2_ presented high humidity resistance in a wide range from 35% RH to 75% RH at room temperature. Interestingly, the sensing performance of 2D UiO‐66‐NH_2_/MoS_2_, particularly its selectivity among six interfering gases and ultralow practical limit of detection (LOD) at 20 ppb, was consequently strengthened because of the improvement of humidity‐resistant properties. In addition, the NO_2_ sensing mechanism of 2D UiO‐66‐NH_2_/MoS_2_ under high RH atmosphere was systematically investigated and explicated using Kelvin probe force microscopy (KPFM) and Raman spectroscopy.

## 2 Results and Discussion

**FIGURE 1:**
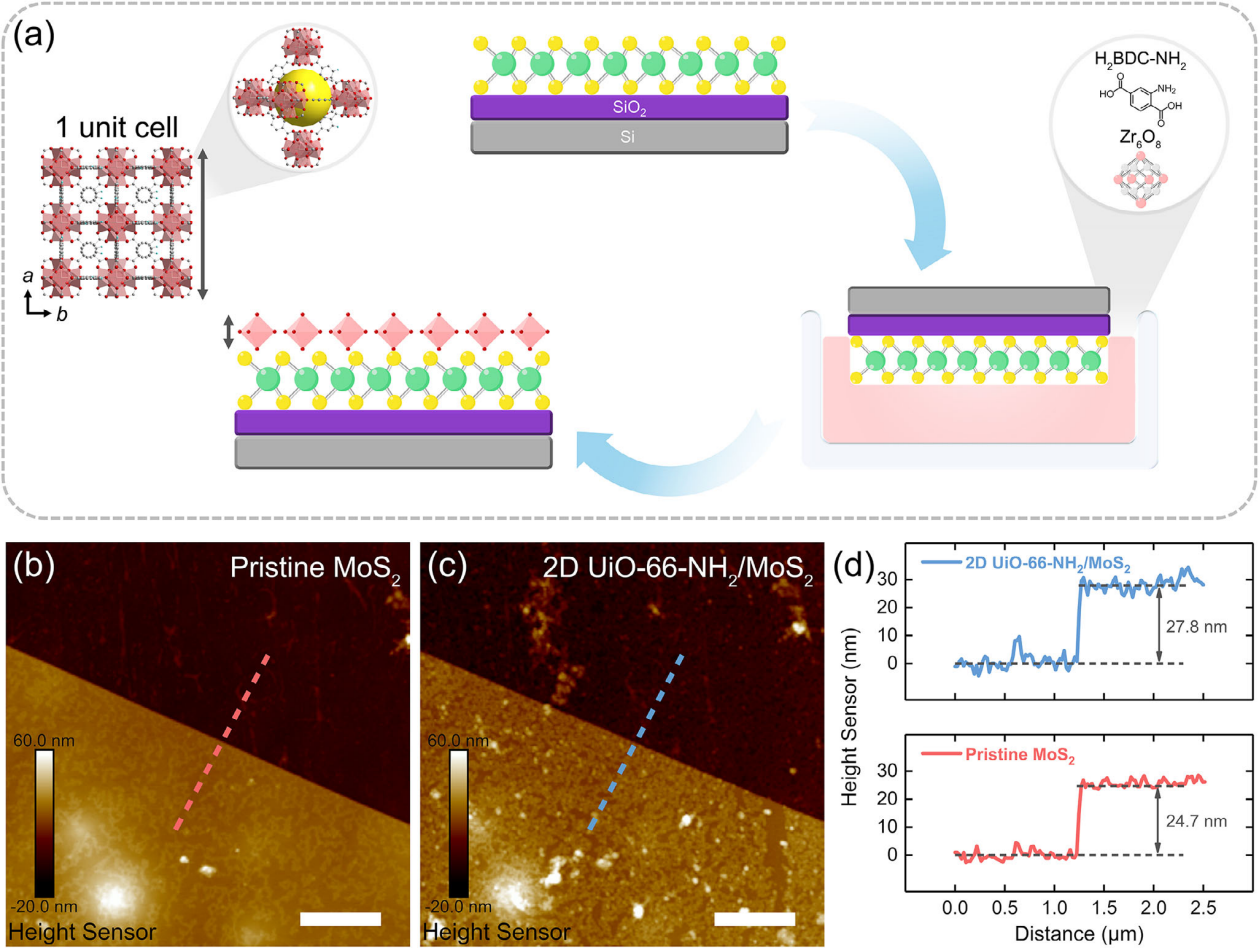
Design and characterizations of 2D UiO‐66‐NH_2_/MoS_2_. (a) Schematic illustration of the fabrication process of 2D UiO‐66‐NH_2_/MoS_2_ via aqueous synthesis. AFM images of (b) pristine MoS_2_ and (c) 2D UiO‐66‐NH_2_/MoS_2_. The scale bars are 1 µm. (d) Corresponding height profiles of pristine MoS_2_ (bottom panel) and 2D UiO‐66‐NH_2_/MoS_2_ (upper panel) along with the dashed lines in (b) and (c), respectively.

2D UiO‐66‐NH_2_/MoS_2_ was fabricated via an aqueous synthesis based on previous work with modifications (details see Supporting Information) [28]. Briefly, benefiting from the flat surficial morphology of the mechanical exfoliated MoS_2_, 2D UiO‐66‐NH_2_ would in situ grow onto its surface by van der Waals interactions at the liquid‐solid interface (Figure 1a). Compared with other surface deposition methods such as dip‐coating or lift‐off technique in the reported works [25, 26], surface modification of MOF materials through in situ van der Waals interactions not only exhibited stronger cohesion but also maintained the integrity and conductivity of 2D materials, whose electronic properties and gas sensing performances could be minimally affected. The MoS_2_ surface morphologies before and after integrating with 2D UiO‐66‐NH_2_ were investigated using atomic force microscopy (AFM). The thickness of the pristine MoS_2_ was about 24.7 nm (Figure 1b), while that of the 2D UiO‐66‐NH_2_/MoS_2_ was about 27.8 nm (Figure 1c), indicating that the thickness of the 2D UiO‐66‐NH_2_ was about 3.1 nm (Figure 1d). According to the literature, the as‐synthesized 2D UiO‐66‐NH_2_ layer was approximately 1 unit cell [28]. Moreover, the encapsulation of 2D UiO‐66‐NH_2_ onto MoS_2_ surface increased its root‐mean‐square roughness from 4.89 to 6.34 nm (Figure S1). The pronounced roughness was thus enhanced the hydrophobicity, which was conducive to improving the humidity resistance of 2D UiO‐66‐NH_2_/MoS_2_ [29, 30]. To verify the successful synthesis of 2D UiO‐66‐NH_2_, the precipitation was collected and dried for the X‐ray diffraction (XRD) and Fourier transform infrared spectroscopy (FT‐IR). Further analysis of XRD showed a (111) plane in contrast with the simulated powder pattern of UiO‐66‐NH_2_ confirmed a lower crystallinity of 2D UiO‐66‐NH_2_ prepared by aqueous synthesis than that prepared by conventional methods (Figure S2), which was in good agreement with the literature [28]. For FT‐IR spectra, two characteristic peaks at 3458 and 3368 cm^−1^ were attributed to the asymmetric and symmetric vibrations of the N─H bond in the amino group, respectively [31]. The strong peaks at 1569 and 1385 cm^−1^ were assigned to the asymmetric and symmetric COO^−^ stretching vibrations in the organic linker [32]. A Peak at 660 cm^−1^ was ascribed to the Zr‐O_2_ vibration in 2D UiO‐66‐NH_2_ (Figure S3) [31]. These results confirmed the successful encapsulation of 2D UiO‐66‐NH_2_ on MoS_2_.

**FIGURE 2:**
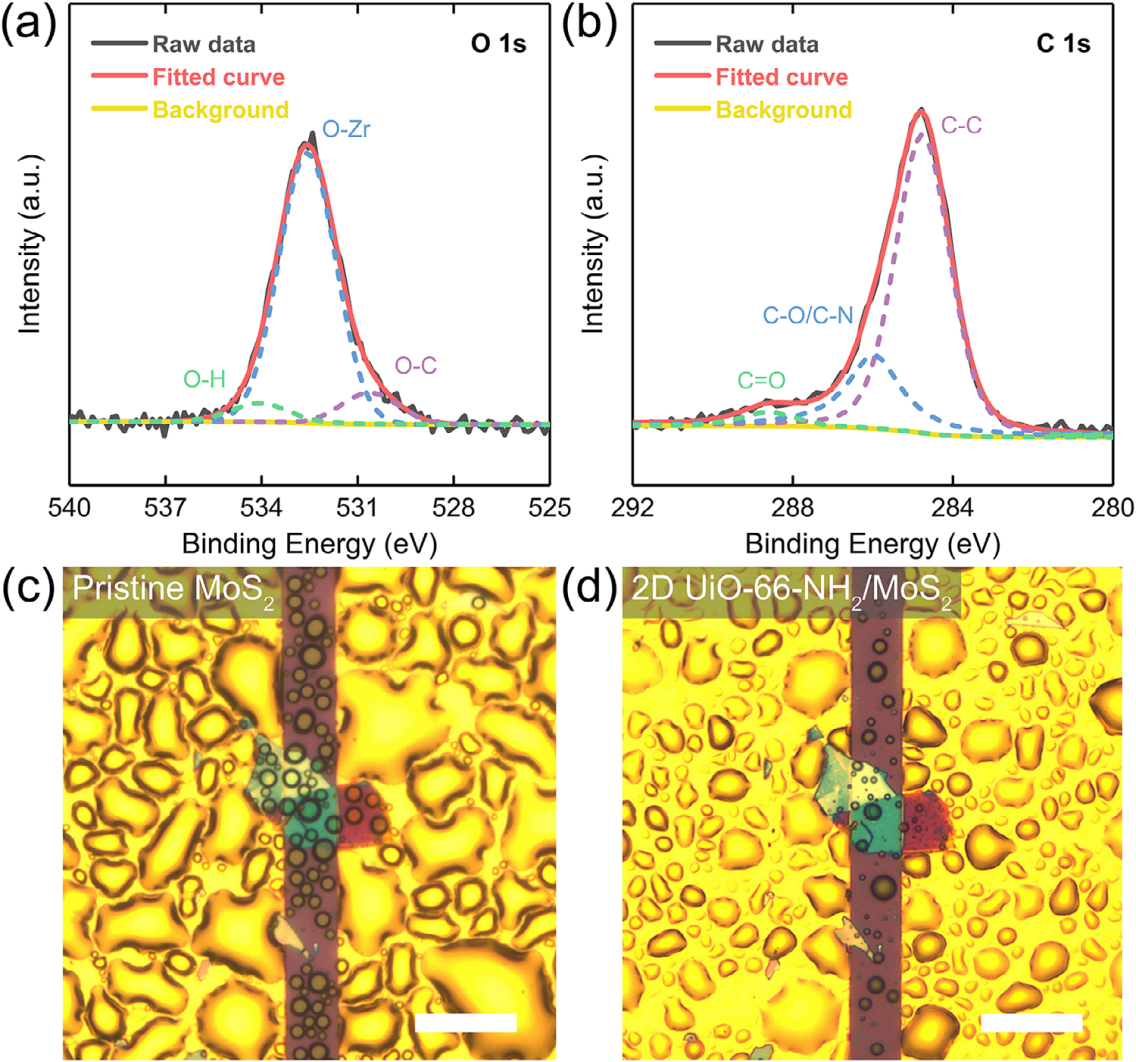
Characterizations of pristine MoS_2_ and 2D UiO‐66‐NH_2_/MoS_2_. High‐resolution XPS spectra of (a) O 1s and (b) C 1s for 2D UiO‐66‐NH_2_/MoS_2_ on Cr/Au electrodes. Optical images of water condensation on the surfaces of (c) pristine MoS_2_ and (d) 2D UiO‐66‐NH_2_/MoS_2_. The scale bars are 40 µm.

The chemical composition of the 2D UiO‐66‐NH_2_/MoS_2_ was analyzed through X‐ray photoelectron spectroscopy (XPS). The survey spectrum identified the integration between 2D UiO‐66‐NH_2_ and MoS_2_ (Figure S4a–c). The O 1s spectrum peaks at 530.6, 532.5, and 534.2 eV were associated with O─C, O─Zr, and O─H groups, respectively (Figure 2a) [33, 34, 35]. The C 1s signals at 284.8, 286.1, and 288.7 eV corresponded to C─C, C─O/C─N, and C═O bonds originating from 2D UiO‐66‐NH_2_, respectively (Figure 2b) [28, 35]. In addition, The Zr 3d spectrum deconvoluted into two peaks at 185.1 eV of Zr 3d_3/2_ and 182.7 eV of Zr 3d_5/2_ (Figure S4d) [36]. These results indicated that the chemical environments of O, C and Zr within 2D UiO‐66‐NH_2_/MoS_2_ were similar to those in bulk crystal. Owing to the nanosheets being too small to measure their water contact angle, a water condensation test was designed to evaluate the hydrophobicity of MoS_2_‐based gas sensing materials in terms of the ubiquitous physical phenomenon, which atmospheric moisture nucleated into liquid condensates upon contact with a sufficiently cold surface [37]. It is obvious that the water almost completely covered the pristine MoS_2_ surface (Figure 2c). After MOF encapsulation, both the amount and area of water condensation on MoS_2_ were significantly reduced (Figure 2d), suggesting that the introduction of 2D UiO‐66‐NH_2_ effectively enhanced the water‐resistant capability of the MoS_2_ surface. Based on the roughness increment of 2D UiO‐66‐NH_2_/MoS_2_ (Figure S1), the water condensation behavior on its surface was consistent with Cassie‐Baxter model [38]. However, as for micro‐nano interfaces, there were various impregnation states because of the different wetting difficulties in micron and nanoscales [39]. Besides, the water spreading on the 2D UiO‐66‐NH_2_/MoS_2_ surface illustrated a full Wenzel regime. Therefore, the Cassie‐Baxter equation required modifications in the existence of a full Wenzel state:

(1) $$\cos\theta_{\mathrm{fw}}=\left(r_{m}+r_{n}-1\right)\cos\theta_{Y}$$

where *θ_fw_* refers to the contact angle under the full Wenzel state, *r_m_* and *r_n_* represent the roughness at the micron scale and nano scale, respectively, and 𝜃*_Y_* is the Young's contact angle between the tangent line of the water drop surface and the horizontal plane. These results explained the improvement of the humidity resistance of MoS_2_ after in situ growth of 2D UiO‐66‐NH_2_.

**FIGURE 3:**
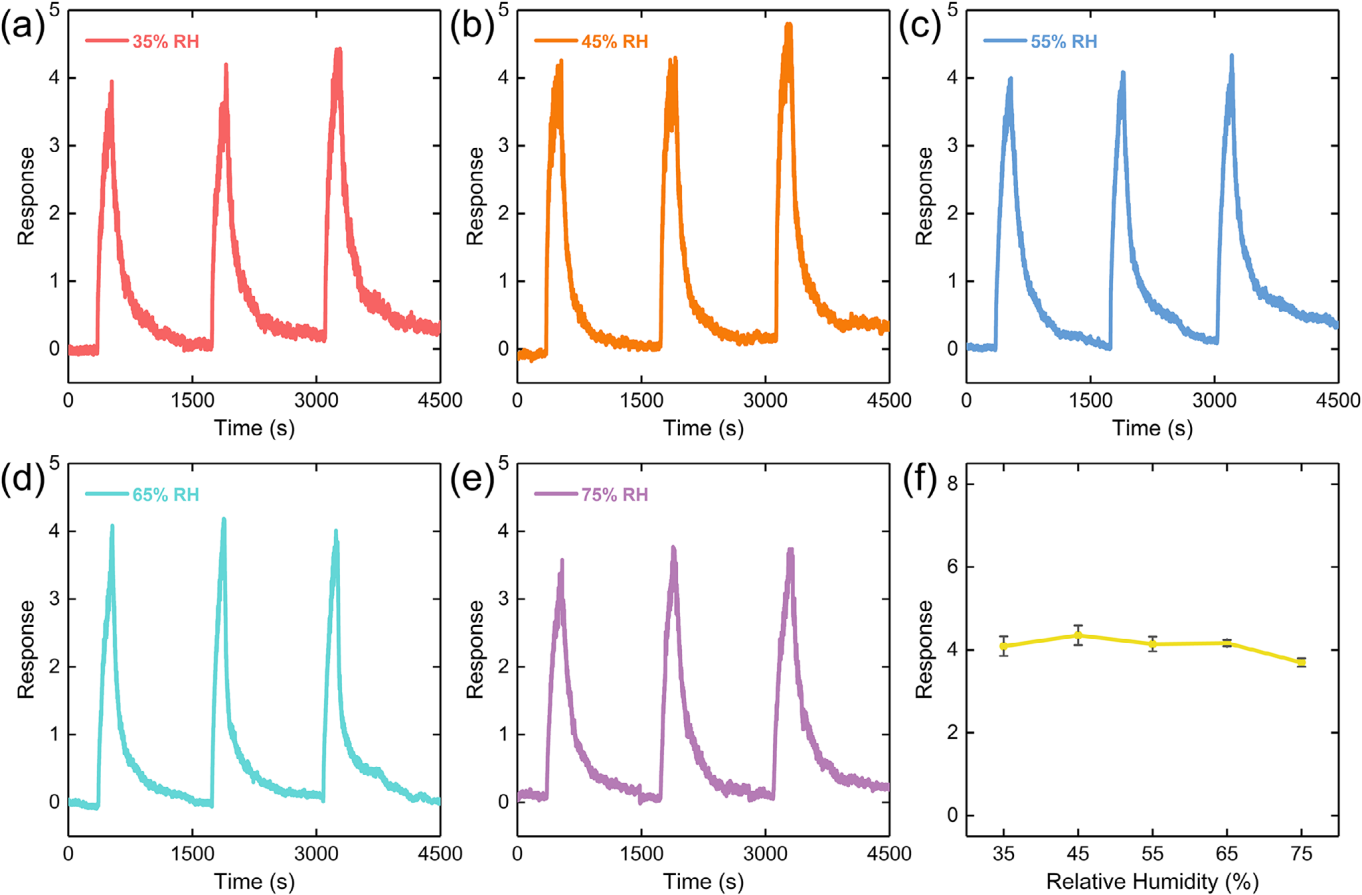
Humidity‐resistant gas sensing performance of 2D UiO‐66‐NH_2_/MoS_2_. 3‐cycle response curves toward 500 ppb NO_2_ at room temperature under (a) 35%, (b) 45%, (c) 55%, (d) 65% and (e) 75% RH, respectively. (e) Corresponding responses of 2D UiO‐66‐NH_2_/MoS_2_ to 500 ppb NO_2_ in the RH range of 35% to 75%. All error bars are plotted in grey and represent standard deviation of three independent experiments.

Given that the outstanding water resistance of the 2D UiO‐66‐NH_2_/MoS_2_ surface, the humidity resistance of the gas sensors were thoroughly investigated. To facilitate the recovery behavior of the gas sensors exposed to the target gases, all the gas sensing measurements were conducted under the 405 nm illumination with a power density of 75 µW/cm^2^. The gas sensing response was defined as:

(2) $$Response=\frac{\left|R_{g}-R_{a}\right|}{R_{a}}\;$$

where *R_g_* and *R_a_* represent the resistance of the gas sensor in the presence of target gases and in the air, respectively. Compared with pristine MoS_2_, in the wide RH range from 35% to 75%, 2D UiO‐66‐NH_2_/MoS_2_ exhibited stable sensing performances toward 500 ppb NO_2_ at room temperature (Figure S5). Under each specific RH environment, the 2D UiO‐66‐NH_2_/MoS_2_ also showed a superior reversible dynamic sensing behavior, indicating its highly reliable reversibility (Figure 3a–e). Particularly, the NO_2_ response under 75% RH still presented at about 3.7 averagely (Figure 3e), showing only 12% decline in contrast with that under 35% RH (average response was about 4.2, Figure 3a). Besides, the response relative standard deviation (RSD) of 2D UiO‐66‐NH_2_/MoS_2_ to the same NO_2_ concentration was about 5.89% (Figure 3f), demonstrating a highly stable, humidity‐resistant NO_2_ sensing performance across a wide RH range of 35% to 75%. Moreover, the comparative gas sensing performances of 2D UiO‐66‐NH_2_/MoS_2_ with and without 405 nm light illumination under humid conditions demonstrated the humidity resistance primarily originated from the MOF encapsulation (Figure S6). The relatively stable baseline resistance of 2D UiO‐66‐NH_2_/MoS_2_ also proved the in situ encapsulation of 2D UiO‐66‐NH_2_ layer effectively enhanced the humidity resistance of the gas sensor (Figure S7).

**FIGURE 4:**
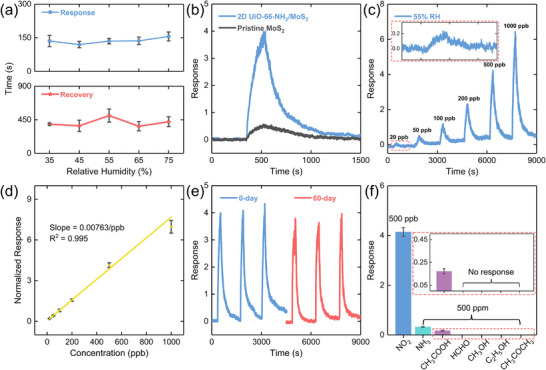
Gas sensing performances of 2D UiO‐66‐NH_2_/MoS_2_. (a) Summaries of response time (upper panel) and recovery time (bottom panel) of 2D UiO‐66‐NH_2_/MoS_2_ under RH ranging from 35% to 75%. (b) Comparative sensing performances of pristine MoS_2_ and 2D UiO‐66‐NH_2_/MoS_2_ toward 500 ppb NO_2_ under 55% RH. (c) Dynamic response of 2D UiO‐66‐NH_2_/MoS_2_ exposed to different NO_2_ concentrations ranging from 20 to 1000 ppb. The inset shows the enlarged sensing curve for 20 ppb NO_2_. (d) Corresponding linear fitting of the response as a function of NO_2_ concentration of 2D UiO‐66‐NH_2_/MoS_2_. (e) Gas sensing stability of 2D UiO‐66‐NH_2_/MoS_2_. 0‐day represents the sensor used for the gas sensing measurement immediately after sensor fabrication, while 60‐day represents the same device used for the gas sensing measurement after 60 days exposure in ambient conditions. (f) Selectivity of 2D UiO‐66‐NH_2_/MoS_2_ to various gases under the same conditions. The concentration of NO_2_ is 500 ppb, while those of all the interfering gases are 500 ppm. All error bars are plotted in grey and represent standard deviation of three independent experiments.

The humidity‐resistant feature of 2D UiO‐66‐NH_2_/MoS_2_ was further investigated from its response and recovery time under various RH conditions. The response time of the sensor ranged from about 120 s to 155 s, while the recovery time ranged from about 390 s to 500 s within the RH range of 35% to 75% (Figure 4a). Especially for the response time, the corresponding RSD under RH variation was only about 3.75%, indicating atmospheric RH would not critically affect the gas absorption on 2D UiO‐66‐NH_2_/MoS_2_ surface. Furthermore, the response of 2D UiO‐66‐NH_2_/MoS_2_ to 500 ppb NO_2_ was about 4.0 under 55% RH, which was more than 7 times compared with that of pristine MoS_2_ (Figure 4b). The response time and recovery time were about 140 s and 500 s, respectively, both faster than those of pristine MoS_2_ (Figure S8). It was interesting that the 2D UiO‐66‐NH_2_ not only served as humidity‐resistant layer but also played a crucial role in improving the sensing performance of MoS_2_. The enhancing mechanism would be discussed in the following section. The dynamic response of 2D UiO‐66‐NH_2_/MoS_2_ toward NO_2_ with concentrations ranging from 20 to 1000 ppb at room temperature showed a continuous increment (Figure 4c). Importantly, compared with the reported NO_2_ sensors, 2D UiO‐66‐NH_2_/MoS_2_ still presented a response of 0.24 toward 20 ppb NO_2_ with an excellent signal‐to‐noise ratio, demonstrating highly competitive NO_2_ sensitivity and highlighting its potential for practice application (Figure 4c inset). To further evaluate its sensing credibility, the theoretical LOD of 2D UiO‐66‐NH_2_/MoS_2_ was calculated as:

(3) $$theoretical\;LOD\;\left(ppb\right)=3\times \frac{\sigma }{slope}$$

where *σ* represents the root‐mean squared deviation of the baseline before NO_2_ exposure and *slope* is obtained from the linear fitting curve of response vs. concentration plot (Figure 4d). The theoretical LOD of 2D UiO‐66‐NH_2_/MoS_2_ sensor was about 8.35 ppb, which was much lower than the practical one, indicating a reliable and ultralow practical LOD achieved in 2D UiO‐66‐NH_2_/MoS_2_. Furthermore, the sensing response increased monotonically with the rising NO_2_ concentration and possessed an excellent linear relationship with R^2^ = 0.995 (Figure 4d). Long‐term stability and selectivity are two additional key criteria for practical gas sensors. In this work, the gas sensing stability of 2D UiO‐66‐NH_2_/MoS_2_ was investigated in detail, revealing an average response of 3.8 after 60 days exposure in ambient conditions and exhibiting only about 8.2% decay relative to the initial value (Figure 4e). Selectivity, one of the most critical parameters for assessing gas sensor performance, was evaluated by recording the resistance changes of the sensors in response to different gases and concentrations. The sensor showed a substantially higher response toward NO_2_ than other gases, including NH_3_, acetic acid, formaldehyde, methanol, ethanol, and acetone, even when the concentrations of all the interfering gases (500 ppm) were 1000 times higher than that of NO_2_ (Figure 4f), indicating 2D UiO‐66‐NH_2_/MoS_2_ was capable of selectively distinguishing NO_2_ from various gases. The extraordinary selectivity of 2D UiO‐66‐NH_2_/MoS_2_ might originate from the amino groups on 2D UiO‐66‐NH_2_ that had strong affinity with NO_2_ through weak van der Waals interactions [40]. Additionally, considering the kinetic diameter of NO_2_ (∼ 3.3 Å), 2D UiO‐66‐NH_2_ possessed an appropriate pore size (∼ 6.0 Å) that allowed NO_2_ molecules to be efficiently sieved by the MOF, resulting in NO_2_ enrichment and subsequently diffusing to the MoS_2_ sensing layer, thereby not only boosting sensitivity but also providing selectivity toward NO_2_ [41, 42, 43].

**FIGURE 5:**
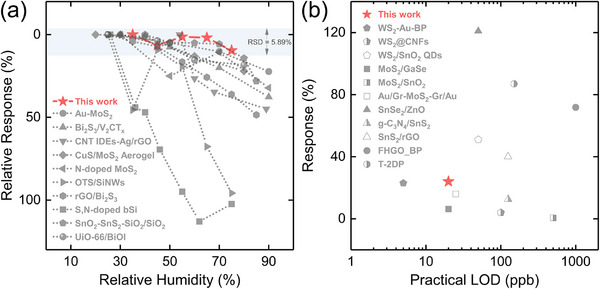
Comprehensive gas sensing performance comparisons of 2D UiO‐66‐NH_2_/MoS_2_ with the reported works. (a) Comparative analysis of RH ranges and relative responses of 2D UiO‐66‐NH_2_/MoS_2_ with the state‐of‐the‐art humidity‐resistant NO_2_ sensors enhanced via surface engineering operated at room temperature. (b) Comparison of responses for 2D UiO‐66‐NH_2_/MoS_2_ with those of previously reported 2D material‐based room‐temperature gas sensors in terms of practical LODs below 1000 ppb.

For intuitively comparing the humidity‐resistant performance of 2D UiO‐66‐NH_2_/MoS_2_ with the reported gas sensors, we herein selected the responses ranged from 20% RH to 80% RH that were close to daily application scenarios, and defined a *Relative response* (%) as:

(4) $$Relative\;response\;\left(\%\right)=\frac{\left|Res_{n}-Res_{1}\right|}{Res_{1}}\;\times 100\%$$

where *Res_n_* and *Res_1_* represent the response of the gas sensors under a specific RH and the first RH (lowest RH) within the range, respectively. Although the testing RH range did not exactly match that of some reported gas sensors, 2D UiO‐66‐NH_2_/MoS_2_ presented excellent stability when RH varied from 35% to 75% with an RSD of 5.89%, ranking among the state‐of‐the‐art gas sensors whose humidity‐resistant properties were enhanced via surface engineering (Figure 5a and Figure S9). Detailed sensing parameter comparisons were summarized in Supporting Information (Table S1). In addition, we also compared the response of 2D UiO‐66‐NH_2_/MoS_2_ with that of the previous 2D material‐based room‐temperature gas sensors at the corresponding practical LOD in ppb level (Figure 5b). It could be seen that 2D UiO‐66‐NH_2_/MoS_2_ demonstrated a relatively satisfied LOD of 20 ppb and response of 24%, which was much lower than the annual average NO_2_ concentration in inhabited areas regulated by the United States Environmental Protection Agency (U.S. EPA) of 53 ppb [44]. The LOD attained by 2D UiO‐66‐NH_2_/MoS_2_ also placed a leading level among the reported 2D material‐based room‐temperature gas sensors, with decent signal‐to‐noise ratio and response. Other gas sensing performances were listed in Supporting Information (Table S2). Therefore, with its high reliability, significantly lower LOD, superior linearity and selectivity, 2D UiO‐66‐NH_2_/MoS_2_ has proven to be an excellent humidity‐resistant NO_2_ sensor at room temperature so far.

**FIGURE 6:**
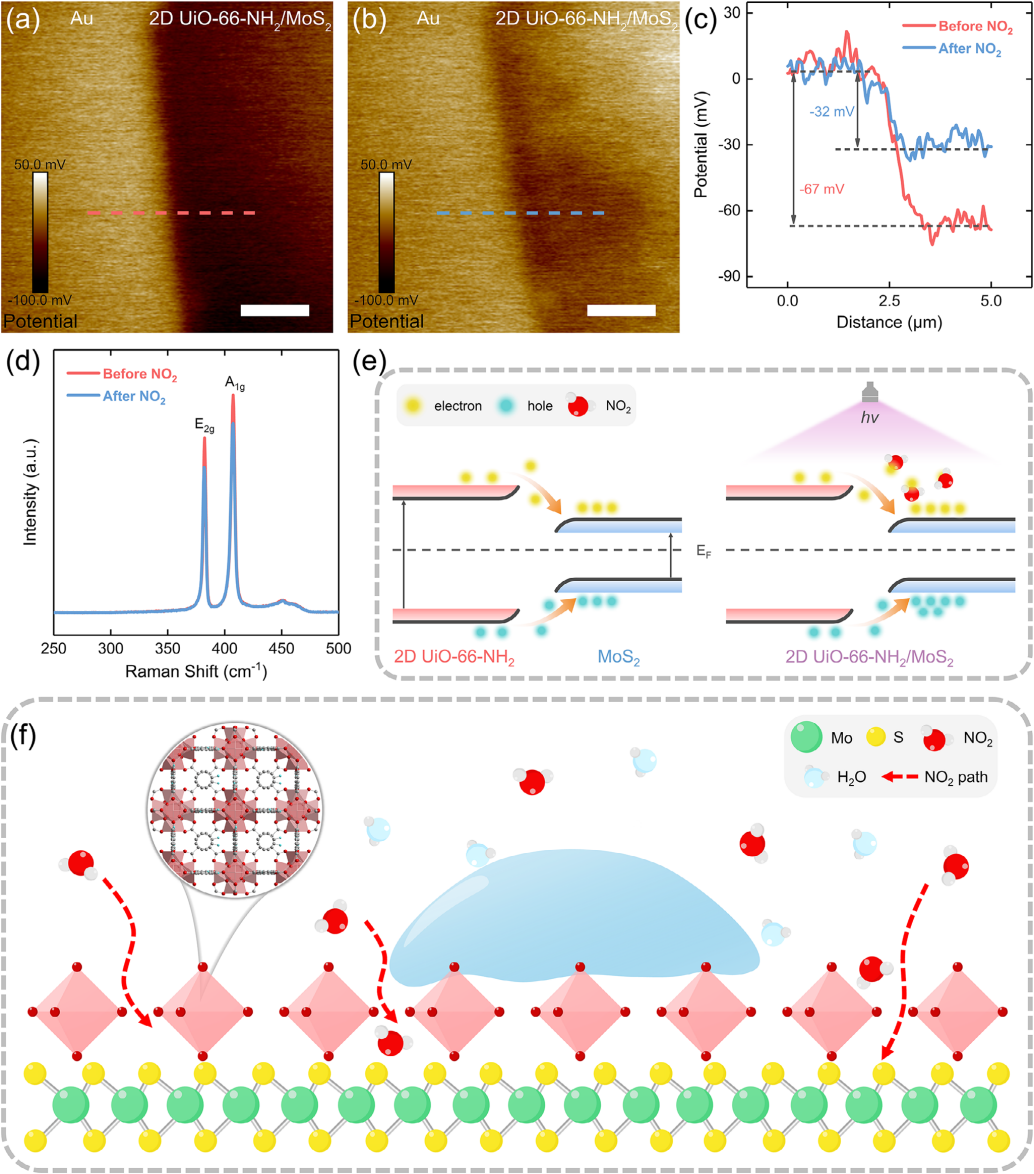
Gas sensing mechanism of 2D UiO‐66‐NH_2_/MoS_2_. KPFM images of 2D UiO‐66‐NH_2_/MoS_2_ (a) before and (b) after NO_2_ exposure. The scale bars are 1 µm. (c) Corresponding surface potential profiles extracted from (a) and (b) along with the dashed lines, respectively. (d) Raman spectra of the 2D UiO‐66‐NH_2_/MoS_2_ before and after NO_2_ exposure at room temperature. (e) Band diagrams of 2D UiO‐66‐NH_2_/MoS_2_ in air (left panel) and under NO_2_ exposure with 405 nm light illumination (right panel). (f) Schematic of the humidity‐resistant NO_2_ sensing mechanism of 2D UiO‐66‐NH_2_/MoS_2_.

Different from the traditional metal oxide‐based gas sensors, the gas sensing mechanism of 2D TMDCs was dominated by the charge transfer between the target gases and sensing materials, which could be experimentally inspected and analyzed by Kelvin probe force microscopy (KPFM) and Raman spectroscopy. First, KPFM was employed to characterize the surface potential of 2D UiO‐66‐NH_2_/MoS_2_ before and after NO_2_ exposure, which was also known as the contact potential difference (CPD) determined by the work function difference between the sample (*W_sample_*) and the AFM probe tip (*W_tip_*):

(5) $$\mathrm{CPD}\;=\frac{W_{sample}-W_{tip}}{e}$$

where *e* represents the elementary charge [16]. Compared with the surface potential of 2D UiO‐66‐NH_2_/MoS_2_ before NO_2_ exposure (Figure 6a), it was obvious that the surface potential of the material sharply increased after contacting with NO_2_ (Figure 6b). Specifically, the surface potential profiles were measured along the dashed lines and the Au substrate was set as reference (Figure 6c). The surface potential of 2D UiO‐66‐NH_2_/MoS_2_ increased from −67 to −32 mV after NO_2_ exposure, indicating the Fermi level (E_F_) shifted toward the valence band. This result suggested that the electrons were transferred from 2D UiO‐66‐NH_2_/MoS_2_ to NO_2_ molecules, corresponding to hole doping for 2D UiO‐66‐NH_2_/MoS_2_, which was consistent with the electron transfer process predicted by the first‐principles calculations [45]. The NO_2_ sensing mechanism was further investigated by Raman spectroscopy at room temperature (Figure 6d). The Raman shifts of the in‐plane (E_2g_) and out‐of‐plane (A_1g_) vibrational modes in MoS_2_ region remained unchanged after NO_2_ exposure in humid conditions, suggesting no observable chemical bonding or structure distortion occurred. However, the intensities of E_2g_ and A_1g_ both exhibited distinct reduction after being exposed to NO_2_, which could be attributed to the phonon self‐energy renormalization and weakening of the phonons [46]. This phenomenon revealed that NO_2_, as an electron acceptor, was physically absorbed on the surface of 2D UiO‐66‐NH_2_/MoS_2_ and induced a charge extraction process from the material [16, 47]. Hence, the NO_2_ sensing mechanism of 2D UiO‐66‐NH_2_/MoS_2_ could be explained (Figure 6e): The bandgap energies of 2D UiO‐66‐NH_2_ and MoS_2_ were approximately 2.85 and 1.30 eV, respectively [48, 49]. When these two isolated materials were brought into contact, the difference in their E_F_ position induced interfacial charge redistribution, leading to the bending in both the conduction and the valence band at the interface. As a result, the electrons and holes in 2D UiO‐66‐NH_2_ tended to transfer to and accumulate in the conduction band and valence band of MoS_2_, respectively, establishing an interfacial equilibrium state. A type I heterojunction was therefore established at their interface, which lead to the efficient charge transfer for NO_2_ sensing (left panel). Upon visible light illumination, 2D UiO‐66‐NH_2_ was excited to generate electrons and holes while MoS_2_ acted as an electron acceptor, inhibiting the recombination of the photogenerated carries in 2D UiO‐66‐NH_2_. Therefore, the concentration of electrons would decrease since the NO_2_ possessed a strong electron affinity and can easily capture electrons from the conduction band of the materials (right panel) [50]. The resistance of 2D UiO‐66‐NH_2_/MoS_2_ then dramatically increased upon NO_2_ exposure, giving a rise to the gas sensing signal. Overall, the humidity‐resistant gas sensing mechanism of 2D UiO‐66‐NH_2_/MoS_2_ could be described as follows: On the one hand, though the water molecules exhibited a kinetic diameter of ∼ 2.68 Å that smaller than the pore size of 2D UiO‐66‐NH_2_ [51], the MOF encapsulated layer could still serve as an effective hydrophobic barrier to restrict the adsorption and condensation of water under humid conditions. Owing to its enhanced hydrophobicity achieved by the nanoscale construction on MoS_2_ surface, 2D UiO‐66‐NH_2_ subsequently prevented the water molecules from occupying the gas sensing active sites of MoS_2_. On the other hand, benefiting from the adequate pore size (∼ 6.0 Å) of 2D UiO‐66‐NH_2_ and the partially uncovered regions of MoS_2_, NO_2_ with kinetic diameter of ∼ 3.3 Å could still easily access to the sensing layer through the diffusion pathways, thus ensuring their efficient gas absorption. Once the gas sensor exposed to NO_2_, which has a strong affinity to electrons, it would be absorbed on the MoS_2_ surface, extracting electrons from MoS_2_ and consequently increasing the resistance. Furthermore, the visible light illumination not only increased the concentration of the carriers in 2D UiO‐66‐NH_2_/MoS_2_, but also facilitated the NO_2_ desorption from the surface, which was responsible for the light‐enhanced gas sensing performances of 2D UiO‐66‐NH_2_/MoS_2_ toward NO_2_ (Figure 6f).

## 3 Conclusion

In summary, 2D UiO‐66‐NH_2_/MoS_2_ was in situ fabricated via aqueous synthesis method to improve its humidity‐resistant performance at room temperature. Remarkably, the hydrophobicity of MoS_2_ was substantially enhanced by taking advantage of the exceptional nanoscale morphology of 2D UiO‐66‐NH_2_, which effectively shielded the active sites and carriers from being occupied by water molecules and interfering gases. Accordingly, 2D UiO‐66‐NH_2_/MoS_2_ demonstrated a highly humidity‐resistant NO_2_ sensing performance when the RH altered from 35% to 75%. Moreover, the selectivity and sensitivity of the sensors were simultaneously reinforced, achieving a practical LOD of 20 ppb with a reliable response of 0.24. The gas sensing mechanism was systematically explored by KPFM and Raman spectroscopy, proving that the electron transfer between 2D UiO‐66‐NH_2_/MoS_2_ and NO_2_ molecules dominated in the sensing process. Overall, the surface engineering strategy we proposed provided a promising route for the design of humidity‐resistant gas sensors at room temperature and expected to enable scalable integration of MOFs with wafer‐scale TMDCs devices.

## Conflicts of Interest

The authors declare no conflicts of interest.

## Supporting information




**Supporting File**:advs74614‐sup‐0001‐SuppMat.pdf.

## Data Availability

The data that support the findings of this study are available from the corresponding author upon reasonable request.
