# Supplementary material for: Surface Engineering on Transition Metal Dichalcogenides: In Situ Encapsulation of Metal–Organic Frameworks for Highly Humidity‐Resistant Gas Sensing at Room Temperature
Source: Adv Sci (Weinh). 2026 Feb 28;13(27):e74614. doi: 10.1002/advs.74614 (PMC13170246; doi:10.1002/advs.74614)
Supplement: Supplementary file 1 — Supporting File:advs74614‐sup‐0001‐SuppMat.pdf. [file ADVS-13-e74614-s001.pdf]

Supporting Information

Surface Engineering on Transition Metal Dichalcogenides: *In Situ* Encapsulation of Metal-Organic Frameworks for Highly Humidity-Resistant Gas Sensing at Room Temperature

Junwei Zeng[1], *Weicheng Jiao*[1]*, *You Wang*[3], *Ye Yuan*[4], *Xiaodong He*[1], and *Yue Niu*[2]*

[1]National Key Laboratory of Science and Technology on Advanced Composites in Special Environments, Harbin Institute of Technology, Harbin 150080, P.R. China

[2]Dongguan Key Laboratory of Interdisciplinary Science for Advanced Materials and Large-Scale Scientific Facilities, School of Physical Sciences, Great Bay University, Dongguan 523000, P.R. China

[3]School of Materials Science and Engineering, Harbin Institute of Technology, Harbin 150001, P.R. China

[4]Songshan Lake Materials Laboratory, Dongguan 523000, P.R. China

**Corresponding Authors: Weicheng Jiao, Email: xiaojiao458@163.com*

Yue Niu, Email: niuyue@gbu.edu.cn

Experimental section

Preparation of MoS_2 Nanosheets. MoS_2 nanosheets were prepared from the commercial bulk MoS_2 crystals *via* mechanical exfoliation method. Briefly, the MoS_2 crystal was first covered by a piece of Nitto tape (Nitto Denko, SPV 224P, Japan) and followed by scrupulously peeling off the tape. Then a poly(dimethylsiloxane) (PDMS) viscoelastic stamp (Gel-pak, WF-30-X4, USA) was pasted on the MoS_2 sheets. Eventually, the MoS_2 nanosheets with suitable thickness and lateral size could be obtained on the stamp.

MoS_2 Gas Sensor Fabrication. The selected MoS_2 nanosheets were inspected with an optical microscope and then transferred from the PDMS stamp onto the Cr (5 nm)/Au (60 nm) electrodes pre-patterned on the SiO_2 (280 nm)/Si wafer by an all-dry transfer method to fabricate the MoS_2 gas sensor.

Preparation of UiO-66-NH_2 Precursor Solutions. The preparation of synthesis precursors for UiO-66-NH_2 was adapted with modifications from the reported synthesis method. Specifically, 2.58 g of zirconyl chloride octahydrate ($ZrOCl_2 \cdot 8H_2O$, Sigma Aldrich, 98%, USA) was dissolved in 40 mL of water containing 10 mL of acetic acid (Innochem, GR, China). The resulting Zr^{4+} solution was then heated at 80 °C for 3 hours to undergo hydrolysis, leading to the formation of Zr_6O_8 clusters. After hydrolysis, the solution was diluted to 500 mL, yielding in a 16 mmol/L Zr^{4+} solution. Separately, 1.49 g of 2-aminoterephthalic acid (H_2BDC-NH_2, Sigma Aldrich, 99%, USA) and 0.64 g of

NaOH (Innochem, ≥96%, China) were dissolved in water to obtain a 500 mL 16 mmol/L $H_2BDC-NH_2$ solution.

2D UiO-66-NH_2/MoS_2 Gas Sensor Fabrication. 5 mL of a 2 mmol/L Zr^{4+} solution, obtained by diluting the previously prepared 16 mmol/L Zr^{4+} solution, was mixed with a certain amount of 1.0 mol/L NaOH to adjust the pH to 3.30. Subsequently, 5 mL of a 2 mmol/L $H_2BDC-NH_2$ solution, prepared by diluting the previously prepared 16 mmol/L $H_2BDC-NH_2$, was added to the petri dish at room temperature to achieve a final concentration of 1 mM for both $H_2BDC-NH_2$ and Zr^{4+}. The as-fabricated MoS_2 gas sensor was placed upside down onto the surface of mixed solution and reacted statically for 60 min. Then the device was rinsed by deionized water to remove the unreacted residual precursors. After vacuum drying at 70 °C for 1 h, the 2D UiO-66-NH_2/MoS_2 gas sensor was fabricated.

Characterizations. The AFM and KPFM images were obtained by atomic force microscope (Bruker, Dimension Icon, USA) operated in the tapping mode with SCM-PIT-V2 probes. The optical microscopy images were acquired with a metalloscope (YUESCOPE, YM520TR, China) in reflection mode. The micro-area XPS spectra were obtained using an X-ray photoelectron spectrometer (ULVAC-PHI, PHI GENESIS 500, USA) using an Al Kα excitation source. The XRD pattern was investigated by an X-ray diffractometer operating at 40 kV with Cu Kα radiation at a scanning rate of 0.5°/min. (Rigaku, Smartlab, Japan). The FT-IR spectra were collected *via* an FT-IR spectrometer (Thermo Fisher Scientific, Nicolet iS50, USA). The Raman spectra were

obtained by a Raman microscope (Horiba, LabRAM Odyssey, Japan) under the 532 nm excitation wavelength before and after NO_2 exposure gas in a quartz chamber.

Surface Water Condensation Test. For proving the hydrophobic effect of the MOF, the MoS_2 and 2D UiO-66-NH_2/MoS_2 gas sensors were first cooled down employing liquid nitrogen for 10 s. Then the treated gas sensors were placed under the metalloscope in a moist environment. As the temperature elevates, atmospheric moisture would condense on the surface of the material surfaces for the existence of temperature difference. Hence, the amount and area of the condensed water on the surface could be used to evaluate the hydrophobicity of materials.

Gas Sensing Measurements. All the gas sensing measurements were carried out under 405 nm light illumination with a power density of 75 $\mu W/cm^2$ using a home-built gas sensing system integrated with a light-emitting diode (LED) light source *via* a standard optical fiber. The power density of the LED light source was modulated by a power supplier and calibrated using a silicon photodiode sensor (Thorlabs, USA, S120VC). The gas sensing signals were recorded utilizing a source meter (Tektronix, USA, Keithley 2450). The gas sensing measurements were conducted in the same environment at room temperature (25 °C) under a certain relative humidity ranged from 35% to 75% which were maintained by a dehumidifier.

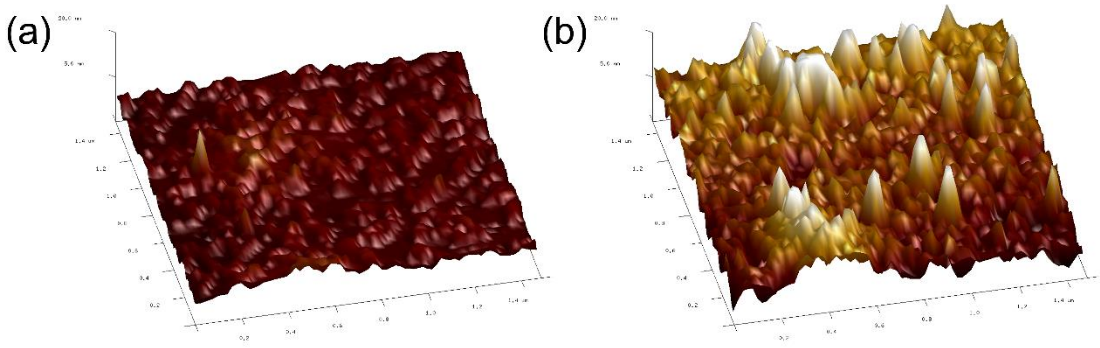

Figure S1. 3D AFM images of (a) pristine MoS_2 and (b) 2D UiO-66-NH_2/MoS_2.

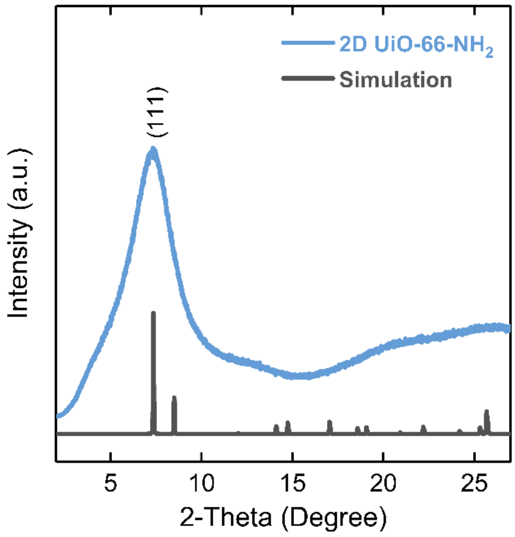

Figure S2. XRD pattern of 2D UiO-66-NH_2.

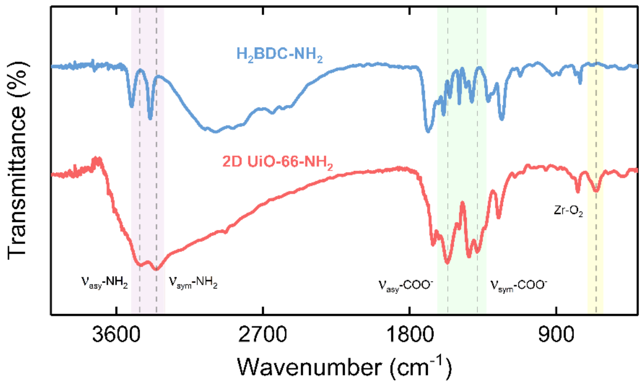

Figure S3. FT-IR spectra of $H_2BDC-NH_2$ (blue) and 2D UiO-66-NH_2 (red).

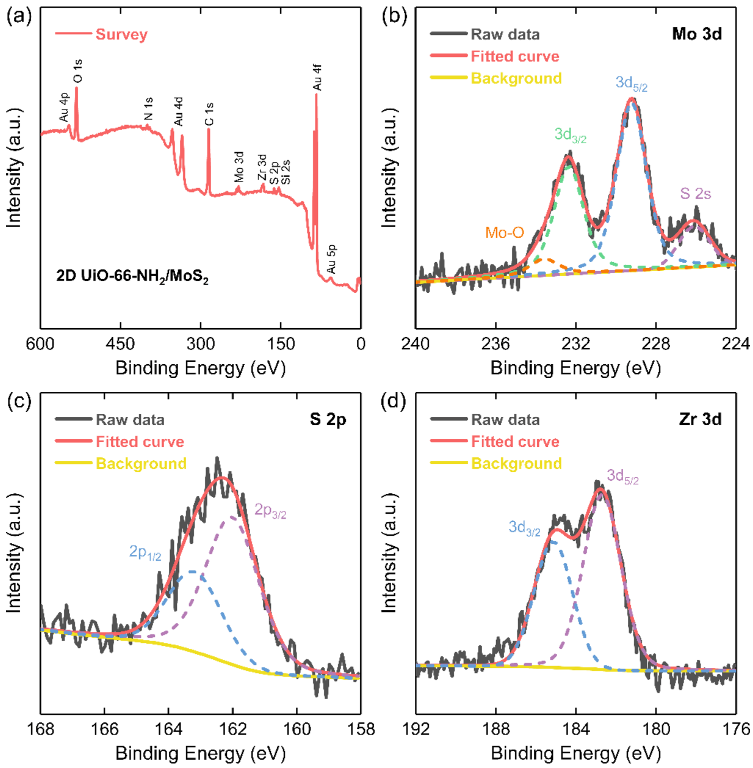

Figure S4. 2D UiO-66-NH_2/MoS_2 XPS spectra of (a) survey spectrum, (b) Mo 3d, (c) S 2p, and (d) Zr 3d, respectively. The tested 2D UiO-66-NH_2/MoS_2 sample was placed on Cr/Au electrodes.

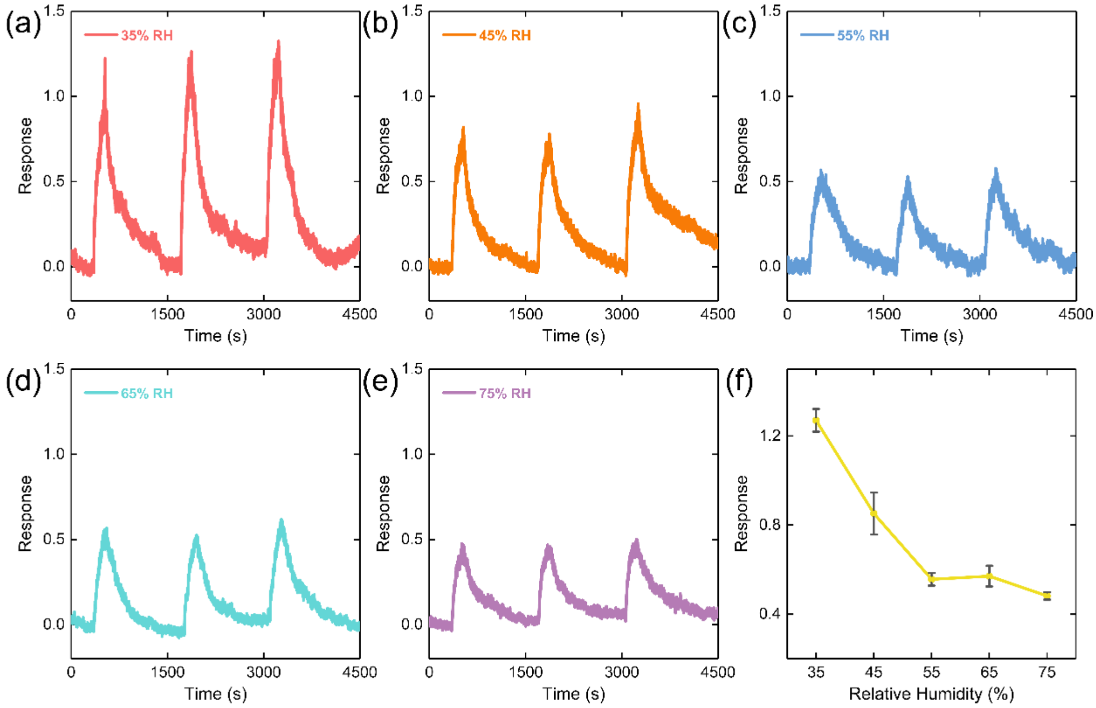

Figure S5. Humidity-resistant gas sensing performance of pristine MoS_2. 3-cycle response curves toward 500 ppb NO_2 at room temperature under (a) 35%, (b) 45%, (c) 55%, (d) 65% and (e) 75% RH, respectively. (e) Corresponding responses of pristine MoS_2 to 500 ppb NO_2 in the RH range of 35% to 75%. All error bars are plotted in grey and represent standard deviation of three independent experiments.

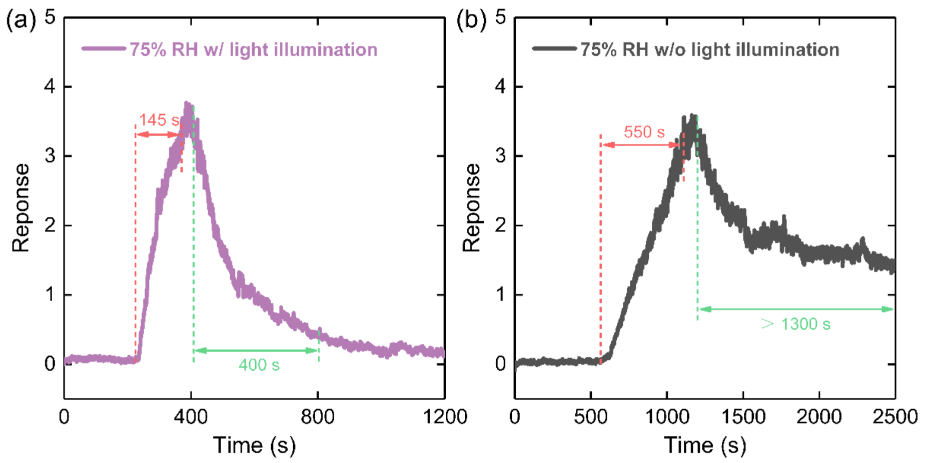

Figure S6. Transient response and corresponding response/recovery time of 2D UiO-66-NH_2/MoS_2 (a) with and (b) without 75 μW/cm^2 405 nm light illumination toward 500 ppb NO_2 at room temperature under 75% RH.

Under the 405 nm light illumination at a power density of 75 μW/cm^2, the response of 2D UiO-66-NH_2/MoS_2 toward 500 ppb NO_2 was about 3.77, while that without light illumination was about 3.59. In addition, the response and recovery time of the sensor under light illumination were 145 s and 400 s, respectively, while those in dark were 550 s and over 1300 s, respectively. It could be concluded that the improvement on the humidity-resistant sensing response was much smaller than that of the response-recovery behavior under light illumination, indicating the improved humidity resistance mainly benefited from the MOF encapsulation layer.

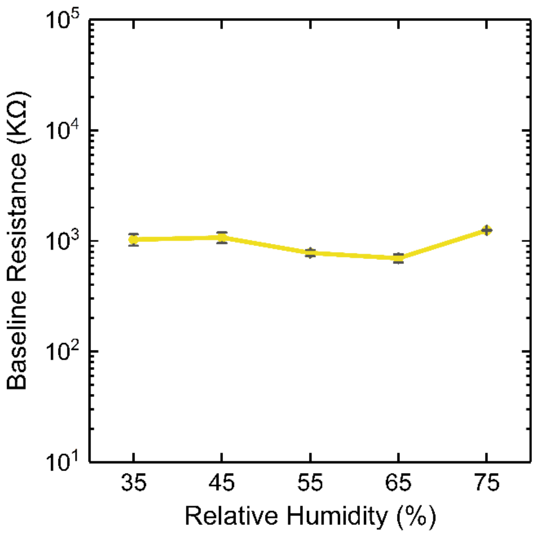

Figure S7. Baseline resistance of 2D UiO-66-NH_2/MoS_2 in the RH range from 35% to 75%. All error bars are plotted in grey and represent standard deviation of three independent experiments.

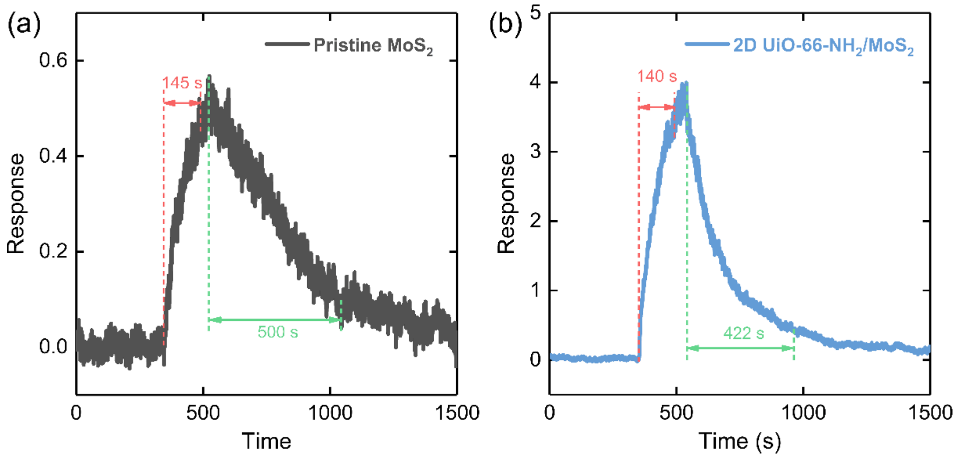

Figure S8. Transient response and corresponding response/recovery times of (a) prsitine MoS_2 and (b) 2D UiO-66-NH_2/MoS_2 toward 500 ppb NO_2 at room temperature.

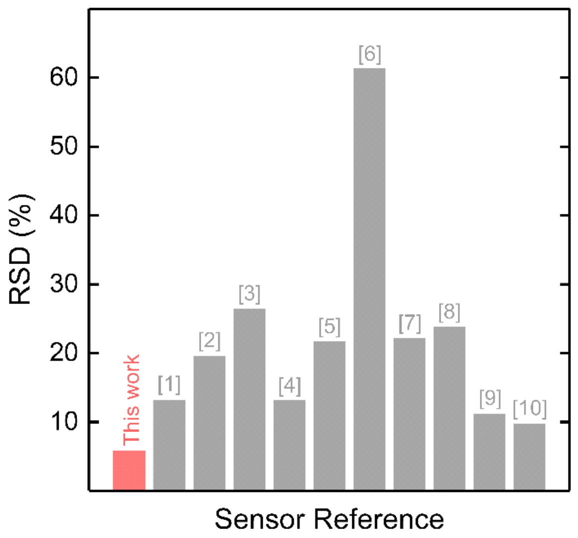

Figure S9. RSD Comparison of 2D UiO-66-NH_2/MoS_2 with the state-of-the-art humidity-resistant NO_2 sensors enhanced *via* surface engineering operated at room temperature. The reference numbers were consistent with Table S1.

Table S1. Comparison of 2D UiO-66-NH_2/MoS_2 with the reported rooom-temperature gas sensors improving their humidity-resistant performance *via* surface engineering.

Materials	RH range	Response range	RSD	Conc.[b]	Res./rec. time[c]	Light	Ref.
Au-MoS_2	10%-90%	4.90-7.08	13.23%	1 ppm	86 s/27 s	530 nm, 10 W	[1]
Bi_2S_3/V_2CT_x	30%-90%	73.1-117.2	19.64%	2 ppm	20 s/103 s	No	[2]
CNT IDEs-Ag/rGO	12%-89%	2.7-5.0	26.5%	20 ppm	220 s/1600 s	No	[3]
CuS/MoS_2 Aerogel	20%-98%	75%-100%[a]	13.17%	20 ppm	89 s/170 s	No	[4]
N-doped MoS_2	10%-90%	0.19-0.35	21.69%	10 ppm	22 s/109 s	No	[5]
OTS/SiNWs	25%-75%	0.018-0.424	61.38%	50 ppb	9 s/185 s	No	[6]
rGO/Bi_2S_3	15%-85%	6.2-10.0	22.21%	1 ppm	22 s/106 s	No	[7]
S,N-doped bSi	26%-75%	6.67-14.20	23.88%	20 ppm	24.8 s/136.8 s	No	[8]
SnO_2-SnS_2-SiO_2/SiO_2	40%-85%	45.3-63.0	11.23%	1 ppm	n.a.[d]	No	[9]
UiO-66@BiOI	25%-80%	37.8-46.3	9.80%	5 ppm	15.3 s/20.3 s	No	[10]
2D UiO-66-NH_2/MoS_2	35%-75%	3.70-4.35	5.89%	500 ppb	140 s/500 s	405 nm, 75 μW/cm^2	*This work*

[a]The response values are presented as relative response by re-calculated from the deviation rate. [b]Conc. represents concerntration. [c]Res./rec. time represrents response/recovery time. [d]Not available.

Table S2. Gas sensing performances comparison of 2D UiO-66-NH_2/MoS_2 with the reported 2D material-based room-temperaure gas sensors with the LOD below 1 ppm.

Materials	Synthesized method	Practical LOD	Response (%)[b]	RH	Res./rec. time (Conc.)	Light	Ref.
WS_2-Au-BP	wet chemistry method	5 ppb	20.3	0%	20 s/103 s (20 ppm)	365 nm, 150mW	[11]
WS_2@CNFs	hydrothermal	100 ppb	4	n.a.	54 s/305 s (10 ppm)	No	[12]
WS_2/SnO_2 QDs	self-assembly	50 ppb	51[c]	n.a.	10 s/9 s (5 ppm)	365 nm, 0.37 mW/cm^2	[13]
MoS_2/GaSe[a]	mechanical exfoliation	20 ppb	6.3[c]	20%	23 s/178 s (500 ppb)	405 nm, 12 mW/cm^2	[14]
MoS_2/SnO_2	wet chemistry method	500 ppb	0.6[d]	n.a.	408 s/162 s (10 ppm)	No	[15]
Au/Gr-MoS_2-Gr/Au	chemical vapor deposition	25 ppb	16	n.a.	n.a	660 nm, 60.9 mW/cm^2	[16]
$SnSe_2$/ZnO	hydrothermal	50 ppb	120.98	0%	747 s/2202 s (50 ppb)	No	[17]
g-C_3N_4/SnS_2	solvothermal	125 ppb	12.6	30%	n.a/1656 s (1 ppm)	No	[18]
SnS_2/rGO	hydrothermal	125 ppb	40[c]	30%	75 s/242 s (1 ppm)	No	[19]
FHGO_BP	air-water assembly	1 ppm	71.8	30%	n.a/n.a (15 ppm)	No	[20]
T-2DP	liquid exfoliation	150 ppb	87[d]	n.a.	35 s/56 s (150 ppb)		[21]
2D UiO-66-NH_2/MoS_2	*in situ* aqueous synthesis	20 ppb	24	55%	140 s/500 s (500 ppb)	405 nm, 75 μW/cm^2	*This work*

[a]Data are collected on self-powered gas sensing mode. [b]Responses are expressed as $\frac{|R_g-R_a|}{R_a} \times 100\%$, unless specified otherwise. R_g and R_a refer to the gas sensors' resistance in the target gas and in the air, respectively. [c]Responses are expressed as $\frac{|I_g-I_a|}{I_a} \times 100\%$, I_g and I_a refer to the gas sensors' current in the target gas and in the air, respectively. [d]Responses are expressed as $\frac{|G_g-G_a|}{G_a} \times 100\%$, G_g and G_a refer to the gas sensors' conductance in the target gas and in the air, respectively.

References

1. P. Chen, J. Hu, M. Yin, W. Bai, X. Chen and Y. Zhang, "MoS_2 Nanoflowers Decorated with Au Nanoparticles for Visible-Light-Enhanced Gas sensing," *ACS Applied Nano Materials* 4, no. 6 (2021): 5981-5991.
2. X. Li, W. Quan, R. Yang, et al., "Bismuth Sulfide Nanorod/Vanadium Carbide MXene as Nitrogen Dioxide Gas Sensor Operating at Room Temperature under Ultraviolet-Assisted Recovery," *ACS Applied Nano Materials* 7, no. 20 (2024): 23882-23893.
3. W. Li, C. Teng, Y. Sun, et al., "Sprayed, Scalable, Wearable, and Portable NO_2 Sensor Array using Fully Flexible AgNPs-All-Carbon Nanostructures," *ACS Applied Materials & Interfaces* 10, no. 40 (2018): 34485-34493.
4. L. Huang, S. Feng, Y. Zhang, et al., "Achieving High Performance of Fuel Cell Type NO_2 Sensor *via* Carrier-Optimized CuS/MoS_2 Aerogel Sensing Electrode," *Sensors and Actuators B: Chemical* 444, (2025): 138429.
5. R. Wu, J. Hao, S. Zheng, et al., "N Dopants Triggered New Active Sites and Fast Charge Transfer in MoS_2 Nanosheets for Full Response-Recovery NO_2 Detection at Room Temperature," *Applied Surface Science* 571, (2022): 151162.
6. Y. Qin, Y. Jiang and L. Zhao, "Enhanced Humidity Resistance of Porous SiNWs *via* OTS Functionalization for Rarefied NO_2 Detection," *Sensors and Actuators B: Chemical* 283, (2019): 61-68.
7. Y. Yang, M. Zhu, H. Zhang, et al., "Room Temperature Gas Sensor Based on rGO/Bi_2S_3 Heterostructures for Ultrasensitive and Rapid NO_2 Detection," *Chemical Engineering Journal* 490, (2024): 151872.
8. W. Wang, H. Li, X. Liu, et al., "Hyperdoping-Regulated Room-Temperature NO_2 Gas Sensing Performances of Black Silicon Based on Lateral Photovoltaic Effect," *Sensors and Actuators B: Chemical* 382, (2023): 133473.
9. J. Liu, J. Zhang, Q. Yu, et al., "Flexible, Breathable and Hydrophobic SnO_2-SnS_2-SiO_2/SiO_2 All-Inorganic Self-Supporting Nanofiber Membrane for Ultralow-

Concentration NO_2 Sensing Under High Humidity," *Advanced Functional Materials* 35, no. 4 (2025): 2410833.

10. H. Chang, J. Fan, R. Chen, et al., "UiO-66@BiOI Core-Shell Structures with High Concentration of Defective Oxygen for NO_2 Gas Sensing at Room Temperature," *Journal of Alloys and Compounds* 1010, (2025): 178327.

11. Z. Liang, M. Wang, X. Zhang, et al., "A 2D-0D-2D Sandwich Heterostructure toward High-Performance Room-Temperature Gas Sensing," *ACS Nano* 18, no. 4 (2024): 3669-3680.

12. Y. Xu, J. Xie, Y. Zhang, et al., "Edge-Enriched WS_2 Nanosheets on Carbon Nanofibers Boosts NO_2 Detection at Room Temperature," *Journal of Hazardous Materials* 411, (2021): 125120.

13. Y. Xia, L. Xu, S. He, et al., "UV-Activated WS_2/SnO_2 2D/0D Heterostructures for Fast and Reversible NO_2 Gas Sensing at Room Temperature," *Sensors and Actuators B: Chemical* 364, (2022): 131903.

14. Y. Niu, J. Zeng, X. Liu, et al., "A Photovoltaic Self-Powered Gas Sensor Based on All-Dry Transferred MoS_2/GaSe Heterojunction for ppb-Level NO_2 Sensing at Room Temperature," *Advanced Science* 8, no. 14 (2021): 2100472.

15. S. Cui, Z. Wen, X. Huang, J. Chang and J. Chen, "Stabilizing MoS_2 Nanosheets through SnO_2 Nanocrystal Decoration for High-Performance Gas Sensing in Air," *Small* 11, no. 19 (2015): 2305-2313.

16. T. Pham, G. Li, E. Bekyarova, M. E. Itkis and A. Mulchandani, "MoS_2-based Optoelectronic Gas Sensor with Sub-Parts-Per-Billion Limit of NO_2 Gas Detection," *ACS Nano* 13, no. 3 (2019): 3196-3205.

17. X. Guo, Y. Ding, X. Yang, et al., "2D $SnSe_2$ Nanoflakes Decorated with 1D ZnO Nanowires for ppb-Level NO_2 Detection at Room Temperature," *Journal of Hazardous Materials* 426, (2022): 128061.

18. Q. Sun, J. Hao, S. Zheng, et al., "2D/2D Heterojunction of g-C_3N_4/SnS_2: Room-Temperature Sensing Material for Ultrasensitive and Rapid-Recoverable NO_2 Detection," *Nanotechnology* 31, no. 42 (2020): 425502.

19. Y. Huang, W. Jiao, Z. Chu, et al., "Ultrasensitive Room Temperature ppb-Level NO_2 Gas Sensors Based on SnS_2/rGO Nanohybrids with P-N Transition and Optoelectronic Visible Light Enhancement Performance," *Journal of Materials Chemistry C* 7, no. 28 (2019): 8616-8625.

20. J. S. Jang, H. J. Jung, S. Chong, et al., "2D Materials Decorated with Ultrathin and Porous Graphene Oxide for High Stability and Selective Surface Activity," *Advanced Materials* 32, no. 36 (2020): 2002723.

21. K. Yang, W. Yuan, Z. Hua, Y. Tang, F. Yin and D. Xia, "Triazine-Based Two-Dimensional Organic Polymer for Selective NO_2 Sensing with Excellent Performance," *ACS Applied Materials & Interfaces* 12, no. 3 (2019): 3919-3927.
